# Vertebral Coccidioidomycosis With Craniocervical Junction Instability and Ventral Displacement of C1 in a Pregnant Woman: A Case Report and Literature Review

**DOI:** 10.7759/cureus.84182

**Published:** 2025-05-15

**Authors:** Saúl Andrés Botello Ramírez, José Navarro Gómez, Elizabeth Meza Mata, Alejandro Gallegos Balderas, José Antonio Candelas Rangel, Gabriela Leilaní Cervantes Pedroza, Gervith Reyes Soto, Carlos Castillo-Rangel, Andreina Rosario Rosario, Manuel De Jesus Encarnacion Ramirez

**Affiliations:** 1 Department of Neurosurgery, High Specialty Medical Unit No. 71, Mexican Social Security Institute, Torreón, MEX; 2 Department of Pathology Anatomy, High Specialty Medical Unit No. 71, Mexican Social Security Institute, Torreón, MEX; 3 Department of Traumatology and Orthopedics, High Specialty Medical Unit No. 71, Mexican Social Security Institute, Torreón, MEX; 4 Neurosurgical Oncology, Mexico National Cancer Institute, Tlalpan, MEX; 5 Neurosurgery, 1st of October Hospital, Institute for Social Security and Services for State Workers (ISSSTE), Mexico City, MEX; 6 Medical School, Autonomous University of Santo Domingo (UASD), Santo Domingo, DOM; 7 Department of Neurosurgery, Peoples' Friendship University of Russia, Moscow, RUS; 8 Department of Human Anatomy and Histology, N.V. Sklifosovsky Institute for Clinical Medicine, Moscow, RUS

**Keywords:** cervical instability, coccidioidomycosis, craniocervical junction, decompression, fixation, vertebral

## Abstract

Coccidioidomycosis is an invasive fungal disease caused by *Coccidioides* species, posing a significant risk of disseminated infection. Vertebral involvement, though rare, can lead to instability, epidural abscess, and neurological deficits. In such cases, the integration of antifungal therapy and surgical intervention becomes critical, yet no standardized surgical protocol exists. A 27-year-old pregnant woman presented with four months of progressive axial neck pain, neck stiffness, a left-sided cervical mass, and neurological deterioration consistent with upper cervical myelopathy. Workup revealed vertebral coccidioidomycosis at the craniocervical junction with ventral C1 displacement and spinal instability. Biopsy of the cervical mass confirmed *Coccidioides* infection, and she was started on fluconazole, amphotericin B, and steroids. Due to worsening neurological function (ASIA B) and imaging showing cervical cord compression, occipitocervical fixation with foramen magnum decompression and partial C1 resection was performed. Despite technically successful decompression and rigid fixation, her neurological function remained unchanged postoperatively. She also experienced preterm labor soon after surgery, and the neonate did not survive. The patient was eventually discharged on lifelong antifungal therapy, maintaining American Spinal Injury Association (ASIA) Impairment Scale grade B status at the short-term follow-up.

Vertebral coccidioidomycosis involving the craniocervical junction is a rare but serious entity that can cause rapid neurological decline. While prolonged antifungal therapy remains essential, timely surgical decompression and stabilization are vital when spinal instability or neurological deficits are present. This case underscores the importance of early diagnosis and intervention for improved outcomes in spinal coccidioidomycosis, especially in high-risk patients such as pregnant women.

## Introduction

Coccidioidomycosis, commonly referred to as San Joaquin Valley Fever, affects an estimated 350,000 people in the United States each year. While historically concentrated in endemic regions like Arizona and California, the disease has been expanding its reach, emerging in new hotspots across northern Mexico and selected areas of Central and South America [1]. This underexplored infection results from inhaling airborne spores of two dimorphic fungi, *Coccidioides* (*C.*) *immitis *and *C. posadasii* [2]. Approximately 60% of those infected remain asymptomatic, with the infection resolving on its own and going undetected in clinical settings [2]. However, around 40% of cases manifest as pulmonary disease, ranging from mild flu-like symptoms to more severe pneumonia [3]. Although relatively rare, coccidioidomycosis imposes a significant strain on healthcare systems, as between 0.5% and 2% of cases progress to disseminated disease [3]. Those at highest risk include immunocompromised individuals, such as pregnant women, organ transplant recipients, chronic steroid users, and patients with human immunodeficiency virus (HIV) or chronic renal failure, as well as non-Caucasian populations, particularly African Americans and Filipinos [3-4].

The disease can spread through two primary pathways: the lymphatic system or the bloodstream, with the latter being the most common. Without antifungal treatment, disseminated infection can develop within two months of initial symptoms or, in rare instances, even years later [5]. If left untreated, the mortality rate can be as high as 25%. Although skeletal involvement is not the most frequent form of dissemination (affecting 16.9% of axial sites like the skull, spine, and rib cage, and 15.5% of peripheral sites like the extremities and other bones), it remains one of the more commonly affected areas [6].

Although some patients may exhibit symptoms of a primary infection, the most frequently reported signs of vertebral coccidioidomycosis include radiculopathy, sensory disturbances, progressive paraparesis, and, most commonly, back or neck pain. It is important to recognize that while dissemination is more likely in symptomatic individuals, vertebral osteomyelitis resulting from disseminated coccidioidomycosis can also occur in those without noticeable signs of primary infection [7-8]. Patients with a history of travel to or residence in endemic regions who do not respond to conservative treatment, or who present with back pain, progressive weakness, or other neurological symptoms, should raise strong clinical suspicion for vertebral infection and undergo a thorough evaluation [9].

Spores of *Coccidioides* are capable of surviving in soil for extended periods. However, they can be effectively killed through methods such as autoclaving, incineration, and chemical disinfection with formaldehyde or bleach solutions (sodium hypochlorite). These sterilization techniques are critical in laboratory and clinical settings where spore handling occurs. In endemic zones, public health measures focus on dust suppression and limiting soil disruption to reduce environmental spore aerosolization [5]. Diagnosing vertebral coccidioidomycosis can be complex, but early identification is crucial to prevent the infection from spreading and worsening the patient’s condition [10]. Although inflammatory markers such as C-reactive protein and erythrocyte sedimentation rate are often elevated, they are not specific to vertebral coccidioidomycosis and may be abnormal in various vertebral infections [11]. More useful for initial evaluation are serologic titers for *Coccidioides* species, as they provide relatively rapid results [10]. Elevated immunoglobulin M antibodies (typically appearing 1-3 weeks after symptom onset) or immunoglobulin G antibodies (rising 2-28 weeks post-onset) above a titer of 1:16 suggest disseminated disease, while titers exceeding 1:128 indicate possible bone or joint infection [11-12].

Imaging studies may reveal vertebral erosions, sclerosis, vertebral plate or disc involvement, osteopenia, vertebral body collapse, or paraspinal extension [12]. Typically, both active and necrotic lesions display prolonged T1 signals with contrast enhancement, while active lesions appear hyperintense on T2-weighted sequences [12]. However, MRI findings alone cannot reliably distinguish coccidioidomycosis from vertebral metastases, tuberculosis, or other infectious diseases [13].

Leptomeningeal disease is one of the more common manifestations, often presenting with widespread leptomeningeal enhancement that affects the entire neural axis. Because of this extensive involvement, comprehensive imaging of both the brain and spine is necessary to determine the full extent of the disease [11].

A frequent misconception is that coccidioidal spondylitis is identical to tuberculous spondylitis. Infections like osteomyelitis that distinctly preserve the disc space typically raise suspicion for tuberculosis or mycotic infections [6]. However, *Coccidioides *spinal infections rarely mimic tuberculous spondylitis, as they tend to preserve disc spaces while affecting adjacent vertebral levels [9]. Additionally, the presence of skipped lesions is common, emphasizing the importance of complete neuroaxis imaging [8].

The most frequent radiologic findings include leptomeningeal enhancement and arachnoiditis, with osteomyelitis being the next most common manifestation. Interestingly, osteomyelitis and leptomeningeal disease are rarely observed together, suggesting that different pathological mechanisms may be responsible for each type of infection [14].

## Case presentation

We present the case of a 27-year-old female patient who was admitted to the ICU with axial cervical pain, neck stiffness, a 4-cm left-sided mass in zone IIB of the neck with a four-month history, wasting syndrome, fever, and signs of cervical myelopathy, classified as ASIA B. The patient exhibited decreased muscle strength in the upper limbs (2/5) and lower limbs (3/5) according to the Daniels scale; bilateral Tromner and Hoffman reflexes, left Babinski and clonus signs, hyperreflexia in all four limbs, and progressive motor function deterioration characterized by muscle hypotrophy, ++ spasticity on the Ashworth scale, difficulty swallowing solids and liquids, and respiratory mechanics alterations. She was 21 weeks pregnant, according to her last menstrual period.

Diagnosis and surgical planning

Upon admission to the ICU, a multidisciplinary team from Otolaryngology, Hematology, Gynecology and Obstetrics (which determined fetal viability), and Surgical Oncology assessed the patient. The latter team performed the initial biopsy of a lymph node (Folio QS-169, 22/03/24), which described histological findings consistent with granulomatous lymphadenitis associated with fungal structures with morphology compatible with *Coccidioides* sp. The Infectious Diseases department initiated treatment with fluconazole and liposomal amphotericin B, along with a steroid regimen including methylprednisolone and dexamethasone.

**Figure 1:**
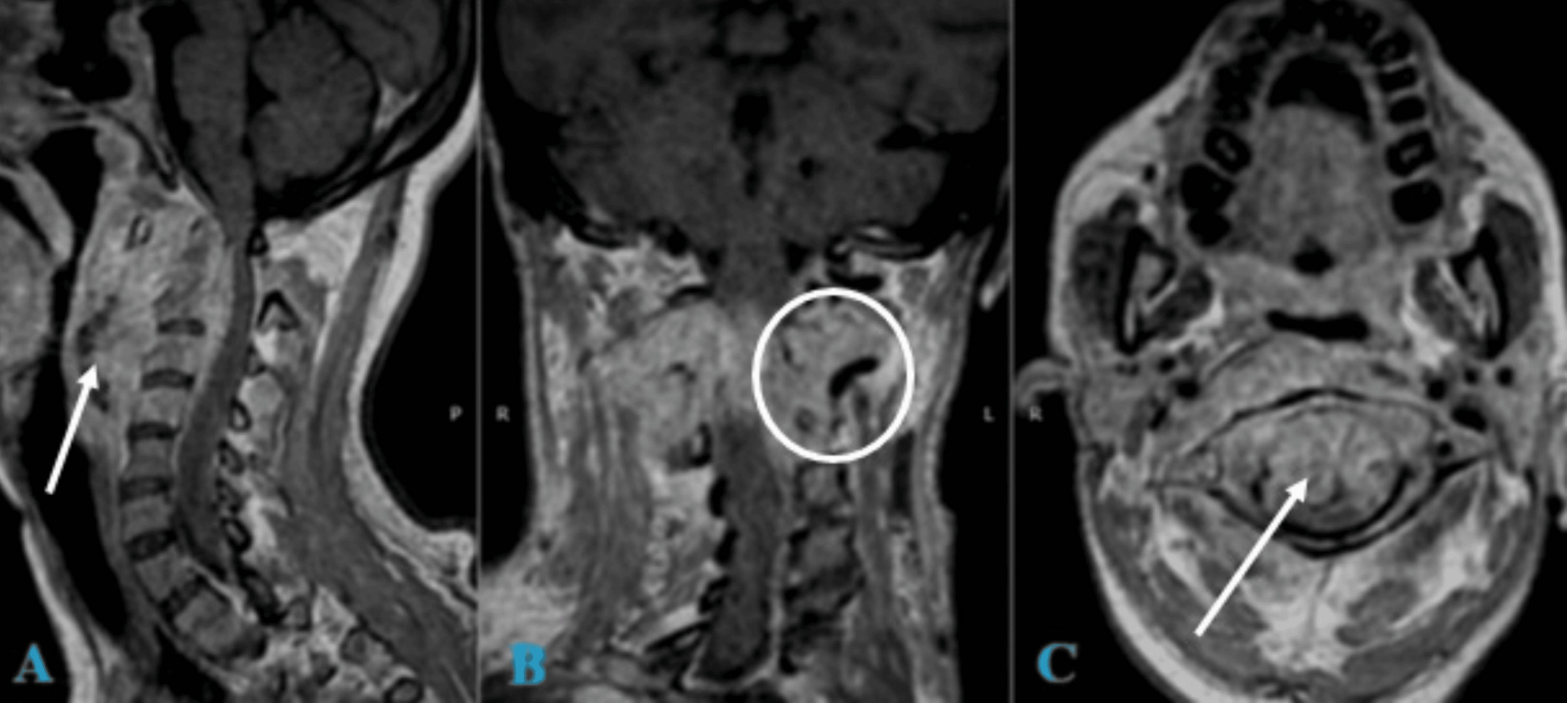
MRI T1 + gadolinium of the cranio-cervical junction and cervical spine A. Sagittal view: Shows a retro-pharyngeal infiltrative process with intradural, extradural, extramedullary invasion and a mass effect causing dorsal displacement of the dural sac, reducing the anteroposterior diameter from C1 to C4. Cervical hyperlordosis of 30.3° is observed. B. Coronal view: Displays infiltration of the lateral masses from C2 to C4 and adjacent soft tissues. C. Axial view at the C1 level: Shows the odontoid process centered within the anteroposterior diameter of C1, with evidence of ventral displacement of C1 and infiltration of the pannus. On the posterior aspect of the odontoid, there is contact with the spinal cord, which is compressed against the ventral surface of the posterior arch of C1, with the spinal cord measuring 4.46 mm in thickness.

**Figure 2:**
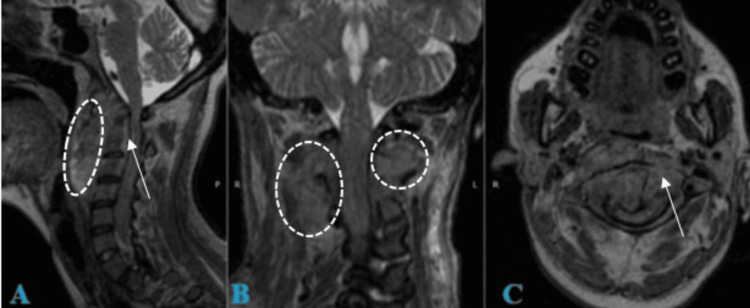
T2 MRI of the cranio-cervical junction and cervical spine A. Sagittal view: Shows a retro-pharyngeal infiltrative process with intradural, extradural, extramedullary invasion. There is an obstruction of cerebrospinal fluid (CSF) flow from the prebulbar cistern and cisterna magna into the cervical dural sac. B. Coronal view: Demonstrates infiltration of the lateral masses from C2 to C4 and adjacent soft tissues. C. Axial view at the C1 level: The odontoid process is centered within the anteroposterior diameter of C1, with evidence of ventral displacement of C1 and pannus infiltration. There is an absence of CSF hyperintensity surrounding the spinal cord.

After four weeks of hospitalization in the ICU under the prescribed treatment plan and preparation of clinical conditions for surgery, spinal cord decompression and occipitocervical fixation were planned based on the following findings from magnetic resonance imaging (Figures 1-2).

Surgical intervention

**Figure 3:**
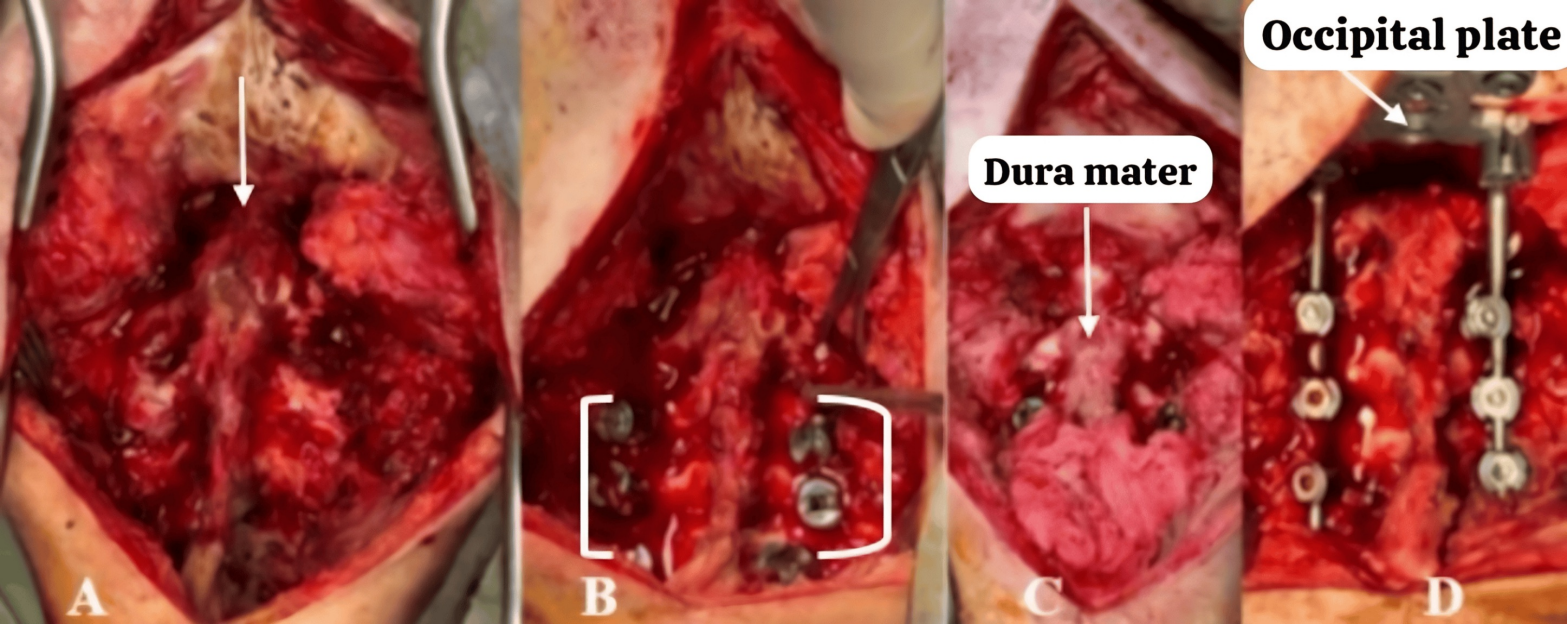
Intraoperative images A. Exposure of the cranio-cervical junction: Shows the spinous processes and lateral masses from C2 to C5. The ventral displacement of the posterior arch is visible. B. Placement of polyaxial screws: Screws were placed from C3 to C5. C. Spinal cord decompression: Drilling of the posterior arch of C1 was performed. The dura mater and free cervical spinal cord are visible at the cranio-cervical junction. D. Placement of occipital plate, rods, and locking screws: Shows the final fixation with the occipital plate and bars in place.

A dissection and exposure of the occipito-cervical junction were performed, followed by drilling and resection of the posterior arch of C1, drilling of the occipital bone, and enlargement of the foramen magnum. A superior C2 hemilaminectomy and C2 flavectomy were conducted, and occipito-cervical arthrodesis with fixation from C3 to C5 using the SOLSTICE system was completed (Figure 3).

**Figure 4:**
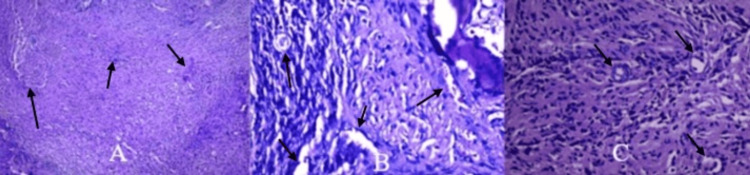
Histopathological study images of the C2 vertebral lesion A. Connective tissue: Shows mild chronic inflammation with chronic fibrosis and multiple non-caseating granulomas. B. Fibrous stroma: Displays chronic inflammation with *Coccidioides immitis* spherules. C. Higher magnification: Shows fibrous stroma with chronic inflammation and a *Coccidioides immitis* spherule.

Intraoperative findings included a ventrally displaced posterior arch of C1 with spinal cord compression. Bone infiltration by coccidioidomycosis was observed in the body and lateral mass of C2 and C3. After decompression, the cervical spinal cord appeared pulsatile. A biopsy was taken from the lateral mass of C2 (Figure 4).

Postoperative evolution

**Figure 5:**
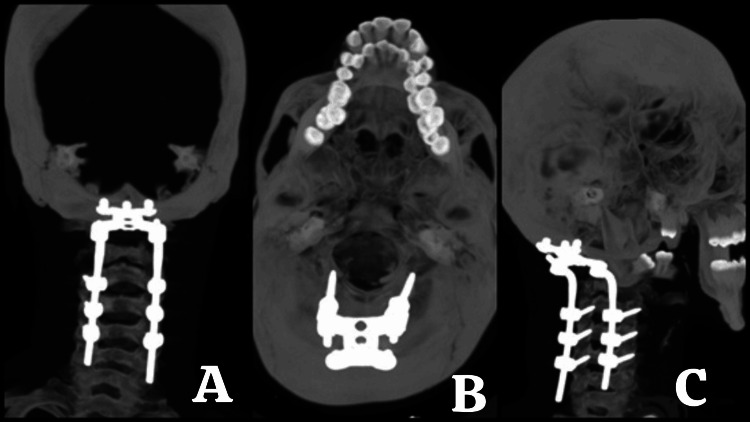
3D reconstruction from skull CT with bone window A. Coronal view showing midline occipital screws and bilateral lateral mass or pedicle screws from C3 to C5 with rods extending caudally. B. Axial view at the craniovertebral junction demonstrating symmetric placement of the occipital plate and screws, confirming proper midline fixation. C. Sagittal view displaying the longitudinal alignment of the occipitocervical construct from the occiput to C6 with maintained anatomical alignment and hardware integrity.

**Figure 6:**
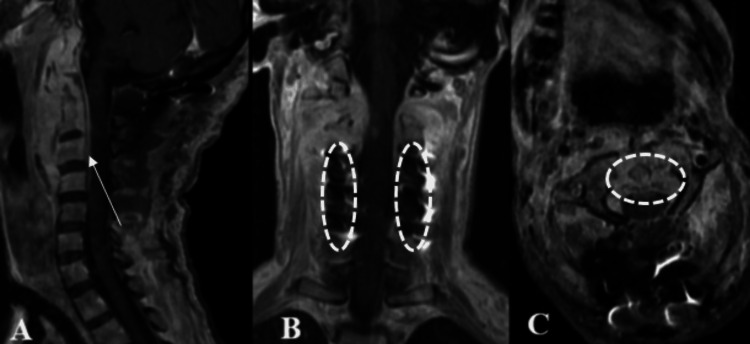
Postoperative T2 MRI A. Sagittal view: Early postoperative MRI following OC fixation shows cervical spine straightening (10°) and decompression at the bulbomedullary junction. Gadolinium enhancement is visible in the retropharyngeal space, indicating postoperative inflammation. Partial reduction of the extramedullary epidural mass is noted. B. Coronal view: Paravertebral hyperintensities are seen at the C2 and C3 levels adjacent to the fixation hardware. C. Axial view at C1 level: The odontoid process appears ventrally repositioned. Hyperintensity surrounds the odontoid and anterior arch of C1, consistent with residual edema and decompression effects.

**Figure 7:**
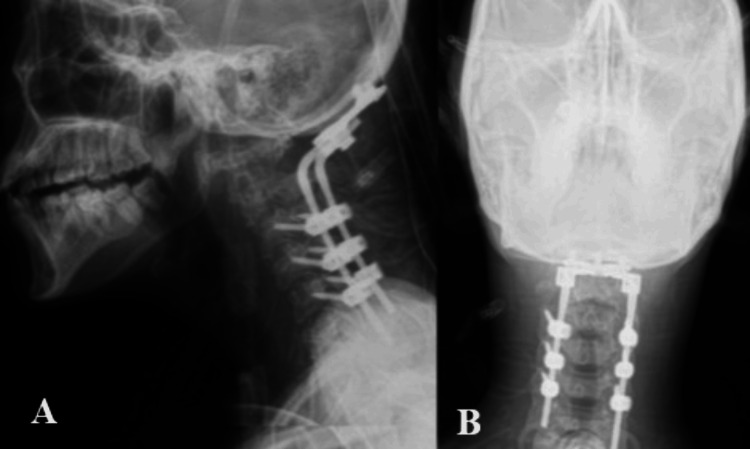
Postoperative X-rays of the cranio-cervical junction A. Lateral view: Postoperative lateral radiograph of the cranio-cervical junction. B. Anteroposterior view: Postoperative anteroposterior radiograph of the cranio-cervical junction.

**Figure 8:**
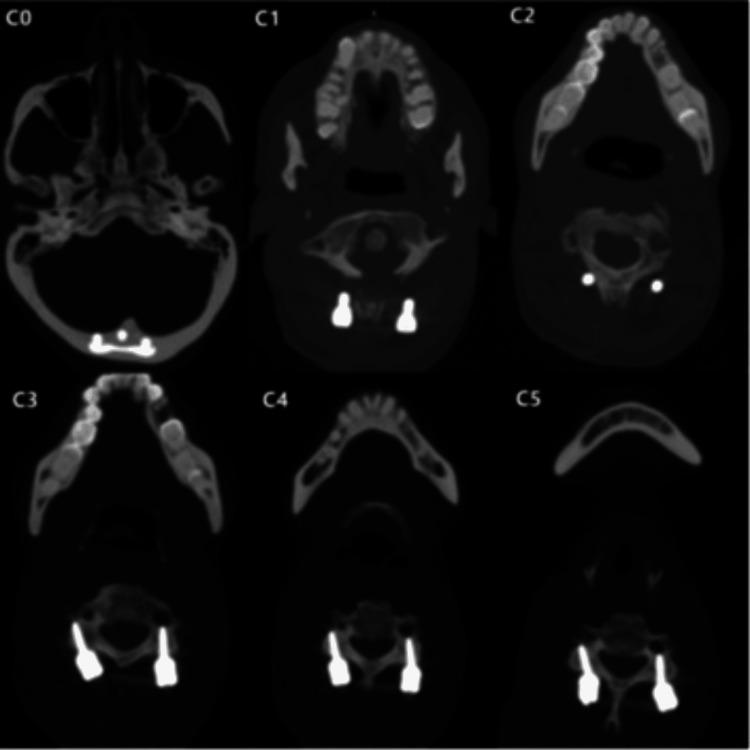
Non-contrast CT of the skull and cervical spine with bone window verifying the entry position of the fixation screws using the Magerl technique.

**Figure 9:**
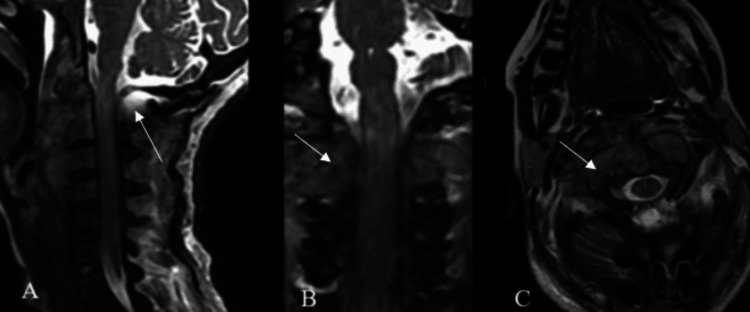
Follow-up postoperative T2 MRI A. Sagittal view: Follow-up MRI at a later postoperative stage shows maintained cervical spine alignment (10°) and sustained decompression at the bulbomedullary junction. B. Coronal view: Craniocaudal hyperintensities extending from C1 to C3 reflect chronic white matter damage from prior compression, now within a decompressed cervical spinal cord. C. Axial view at C1 level: Evidence of surgical decompression, with cerebrospinal fluid (CSF) hyperintensity surrounding the spinal cord. The posterior arch of C1 is absent, consistent with prior surgical drilling.

**Figure 10:**
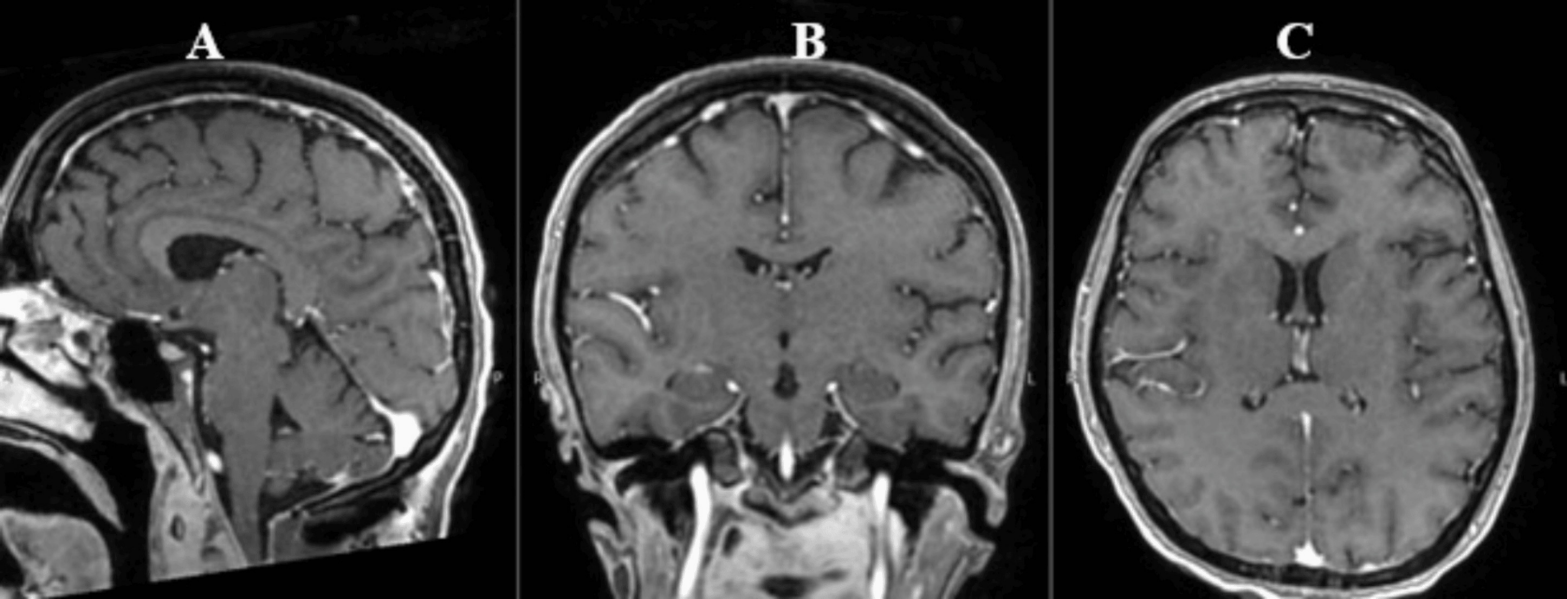
T1 + gadolinium MRI A. Sagittal view B. Coronal view C. Axial view demonstrates the absence of coccidioidomycosis dissemination at the brain level.

The patient continued to experience the neurological deficits described prior to surgery, despite an effective surgical intervention, as demonstrated in subsequent studies (Figures 5-6). She required continued support with a gastrostomy tube and tracheostomy. Additionally, in the immediate postoperative period, she developed preterm labor 24 hours after surgery, and the newborn passed away after a 72-hour stay in the Neonatal Intensive Care Unit (NICU). The patient was discharged home five weeks after surgery, with neurological function classified as ASIA B on the ASIA scale. The following postoperative imaging studies were performed (Figures 5-10).

## Discussion

In this confirmed case of vertebral coccidioidomycosis at the cranio-cervical junction, it is essential to highlight key factors that can benefit patients from surgical treatment during their hospital stay. Determining when medical treatment has truly failed is challenging, as newer azole drugs (e.g., itraconazole and voriconazole) can be effective against resistant coccidioidomycosis [15].

The timing of surgical intervention after the onset of neural function loss is a critical predictor of neural recovery post-surgery in trauma and metastatic diseases. Studies have shown that patients undergoing surgical decompression within 12 hours of a cervical spinal cord injury have better neurological recovery compared to those with delayed intervention. The benefits of surgical decompression decline quickly, even as early as 24 hours post-presentation. In cases of metastatic spinal cord compression, patients who received decompression within 48 hours had improved neurological outcomes compared to those with later surgeries. Conversely, the timeline between the onset of neurological deficits and surgical intervention has not been previously described for spinal and spinal cord infections [15].

Previous large retrospective case series have suggested that medical treatment failure and the presence of neurological deficits, epidural abscess, spinal instability, or intractable pain are valid indications for surgical intervention in spinal coccidioidomycosis. In surgical cases, the most commonly cited indications were spinal instability on imaging, neurological deficits on examination, or spinal cord compression on imaging. Interestingly, the duration of neurological deficit (ranging from one day to two months) did not appear to influence this decision [16,17].

In managing vertebral coccidioidomycosis, particularly cases involving complex anatomical challenges such as craniocervical instability and ventral displacement of the vertebrae, the integration of advanced technologies like exoscopes, 3D printing, and augmented reality (AR) offers promising support for surgical precision and patient outcomes [18]. These technologies allow surgeons to enhance visualization, improve preoperative planning, and refine intraoperative navigation, all of which are critical for managing intricate cases with potential risks to neural structures [19].

The use of exoscopes in spinal surgery has shown significant advantages in terms of visualization and ergonomics, which are essential in cases requiring delicate manipulation around compromised structures, such as the craniocervical junction [18]. Exoscopes provide high-definition magnification, improved lighting, and a wider field of view than traditional microscopes, allowing for a more detailed assessment of affected regions. In this case, an exoscope facilitated precise resection and fixation, contributing to reduced surgical time and potentially minimizing complications [19,20]. Moreover, studies indicate that exoscopes may be particularly beneficial in low-resource environments where traditional surgical microscopes are cost-prohibitive, enabling greater access to advanced neurosurgical care [18].

3D printing technology is transformative for preoperative planning, especially in complex spinal infections where structural deformities may obscure anatomical landmarks. In this case, the ability to create a patient-specific model of the craniocervical junction could aid in simulating surgical steps, predicting potential obstacles, and determining the optimal trajectory for stabilization hardware [21,22, 23]. Printed models enhance the surgical team’s spatial understanding of the affected area and are invaluable for multidisciplinary discussions, enabling more accurate and confident surgical approaches [24]. By visualizing the intricate anatomy involved in vertebral coccidioidomycosis, 3D printing helps in strategizing decompression and fixation, ultimately supporting improved surgical outcomes [25].

Less common indications included medical treatment failure and progressive deformity on imaging. Conversely, the primary reason for not pursuing surgery was the absence of neurological deficits. Overall, the presence of neurological deficits, neural compression on imaging, and spinal instability are the most influential factors in deciding on surgical intervention. The chronic, indolent natural course of spinal coccidioidomycosis may partly explain why patients continue to benefit from surgical decompression even beyond 48 hours from symptom onset [15].

In this case, imaging findings revealed spinal cord edema, often associated with leptomeningeal disease. Coccidioidal spondylitis was also identified and associated with myelopathy, although it is a clinically uncommon presentation [16], as observed in our patient. Vertebral dissemination of coccidioidomycosis has been shown to present as back and neck pain, radiculopathy, sensory disturbances, and motor weakness, and it can also cause discitis, infection of paravertebral soft tissues, vertebral body erosion, and neural compression, all of which were integrated into our diagnostic approach and confirmed with histopathological findings shown in previous images.

Limitations of this study

Single Case Study

This study is based on a single case, limiting the generalizability of findings to broader populations. Without a larger cohort, it is challenging to establish consistent patterns or to determine the reproducibility of treatment outcomes in similar cases of vertebral coccidioidomycosis.

Lack of Long-Term Follow-Up

Due to the short postoperative follow-up, it is not possible to assess the long-term outcomes of both neurological recovery and recurrence of infection. Extended observation would provide a better understanding of the effectiveness of the combined antifungal and surgical treatment over time.

Complexity of Multidisciplinary Care

This case involved multiple specialist teams, including otolaryngology, hematology, obstetrics, and oncology. The multidisciplinary approach adds complexity to care and may not be easily replicable in settings with limited access to specialized healthcare providers, affecting the applicability of this treatment protocol in other settings.

Imaging Limitations

Although MRI and CT imaging provided valuable insights into the spinal pathology, imaging alone could not definitively differentiate coccidioidomycosis from other infectious or malignant conditions. In this study, histopathology was essential for diagnosis, highlighting the limitation of relying on imaging in similar cases.

Impact of Pregnancy

The patient’s concurrent pregnancy presented unique challenges, including increased surgical risk and potential limitations in the choice and duration of pharmacologic interventions. This may affect the applicability of the treatment approach to non-pregnant patients or those with different physiological profiles.

Timing of Surgical Intervention

While the case emphasized the importance of early surgical intervention, the optimal timing of surgery in cases of spinal infections like coccidioidomycosis remains uncertain. The study lacks a comparison with cases where surgery was performed at different intervals, limiting conclusions about timing as a predictor of recovery.

Limited Data on Infection Progression

Coccidioidomycosis can have a chronic and indolent course, and the factors predicting progression to vertebral involvement are not fully understood. This study did not explore the pathogen's progression or the patient's prior exposure history, which could offer insights into risk factors and disease development.

Generalizability of Surgical Indications

Surgical indications in this case were guided by specific clinical findings, such as spinal instability and neurological deficits. The applicability of these indications to a broader population may be limited, as other cases of vertebral coccidioidomycosis may present with differing symptomatology or disease progression.

## Conclusions

In treating vertebral coccidioidomycosis, there is no standardized protocol for surgical intervention. It is essential to determine the appropriate timing of intervention based on clinical evidence of neurological deterioration, continuous monitoring of antifungal treatment effectiveness, and serial imaging studies. While vertebral coccidioidomycosis remission has been documented with antifungal treatment alone, surgical intervention should proceed when urgent factors are present, such as clinical signs of compressive myelopathy, mechanical instability, and intradural infiltrative processes that lead to stenosis, compression, and spinal invasion, as well as neuroinfection risks. The medical community should emphasize that the time between clinical presentation and decompression surgery is the decisive factor for halting or even reversing myelopathy in early cases and reducing morbidity and mortality. New multidisciplinary management algorithms, guided by standardized medical-surgical protocols and functional scales for myelopathy due to infectious processes like vertebral coccidioidomycosis, are necessary to establish optimal surgical timing. Despite technically successful decompression and fixation, as in this case, the outcome may be unfavorable if intervention does not occur at the appropriate time.
